# Antidepressant-Like Effect of *Ilex paraguariensis* in Rats

**DOI:** 10.1155/2014/958209

**Published:** 2014-05-04

**Authors:** Elizete De Moraes Reis, Francisco Waldomiro Schreiner Neto, Vitória Berg Cattani, Luis Ricardo Peroza, Alcindo Busanello, Caroline Queiroz Leal, Aline Augusti Boligon, Tássia Fontana Lehmen, Milena Libardoni, Margareth Linde Athayde, Roselei Fachinetto

**Affiliations:** ^1^Programa de Pós-Graduação em Farmacologia, 97105-900 Santa Maria, RS, Brazil; ^2^Curso de Farmácia, Universidade de Cruz Alta, 98020-290 Cruz Alta, RS, Brazil; ^3^Programa de Pós-Graduação em Ciências Biológicas/Bioquímica Toxicológica, 97105-900 Santa Maria, RS, Brazil; ^4^Curso de Farmácia, Universidade Federal de Santa Maria, 97105-900 Santa Maria, RS, Brazil; ^5^Departamento de Farmácia Industrial, Universidade Federal de Santa Maria, 97105-900 Santa Maria, RS, Brazil

## Abstract

In this study, we investigated the possible antidepressant-like effect of *I. paraguariensis* in rats. Rats were treated for four weeks with an aqueous extract of *I. paraguariensis* in drinking water, following the traditional preparation of this beverage. After the period of treatment, behavioral (elevated plus-maze, open field test, and forced swimming test) and biochemical parameters (lipid peroxidation assay, thiol content, vitamin C levels, and monoamine oxidase activity) were evaluated. Animals were also analyzed on forced swimming test after 24 hours of *I. paraguariensis* intake. An additional group was injected with selegiline 24 hours and 30 minutes before forced swimming test as positive control. HPLC analysis revealed the profile of *I. paraguariensis* extract. *I. paraguariensis* reduced the immobility time on forced swimming test without significant changes in locomotor activity in the open field test. Any anxiolytic/anxiogenic effect of *I. paraguariensis* was observed in rats through the elevated plus-maze test. The antidepressant-like effect of *I. paraguariensis* was not accompanied by inhibitory effect on monoamine oxidase activity. There were no significant alterations on lipid peroxidation, thiol content, and vitamin C levels among the groups. In conclusion, aqueous extract of *I. paraguariensis* decreases the time of immobility in rats suggesting an antidepressant-like effect.

## 1. Introduction

Depression is a psychiatric illness with a high prevalence in humans reaching 21% of the worldwide population [1]. It is well known that pathophysiology of depression involves a dysfunction in monoamine neurotransmitter circuits in the central nervous system [2]. The treatments of depression have monoamines, their receptors and transporters as a target [2]. There are several classes of synthetic drugs available for treating depression in humans: tricyclics, monoamine oxidase inhibitors, selective serotonin reuptake inhibitors, and atypical antidepressants as mirtazapine, for example [3]. However, even the more recent synthetic antidepressants, like atypical ones, have numerous side effects. These undesirable effects compromise the life quality of the patients and, consequently, their clinical use by causing the relapse of the treatment and the recurrence of the symptoms. Furthermore, about 30% of patients do not present remission under therapy with these drugs, which leads to the association of more than one class of antidepressants beyond other classes of drugs, as atypical antipsychotics. All of these pharmacological combinations predispose the patient to severe side effects [4].

Alternative to synthetic antidepressant drugs, the population uses some phytochemical preparations with relative success. Medicinal herbs have been marketed to treat depression [5], such as* Hypericum perforatum* commonly known as St. John's wort.* H. perforatum* has been used for treating mild to moderate forms of depression and its antidepressant effect is attributed to inhibition either of monoamine oxidase (MAO) or serotonin reuptake [6, 7]. Therefore, the search for other natural compounds present in everyday life of the population could be interesting as auxiliary in the antidepressant treatment with fewer side effects than conventional therapy.

In this context,* Yerba mate (Ilex paraguariensis)* is a beverage commonly consumed in South America especially in Argentina, Brazil, Uruguay, and Paraguay. It is a stimulating beverage traditionally consumed as infusion locally known as “chimarrão” or “mate” [8]. Lately, the* I. paraguariensis* has gained rapid penetration into the worldwide markets, either as tea itself or as an ingredient in the industries of food and dietary supplement [9]. The* I. paraguariensis* has a range of biological activities which are attributed to its high polyphenol content. In addition to flavonoids as quercetin and rutin and phenolic compounds as chlorogenic and caffeic acids,* yerba mate* is also rich in caffeine and saponins [10]. The literature data have demonstrated that* I. paraguariensis* can improve the cognition of rats treated with acute administration of hydroalcoholic extract probably through its antagonist's action on adenosine receptors [11]. Another study showed that an infusion of* I. paraguariensis* can improve the memory of rats treated with haloperidol and this effect was related to an indirect modulation of oxidative stress [12]. Oxidative damage is implicated in the pathogenesis of various neuropsychiatric disorders including major depression [13–15]. However, besides innumerous studies about pharmacological properties of* I. paraguariensis*, any study was drawn to investigate if this plant has antidepressant-like effects.

In the present study, we evaluated the possible antidepressant-like effect of* I. paraguariensis* by using forced swimming test (FST) in rats. As a large quantity of antidepressant drugs act through monoamine oxidase inhibition, we investigated if the* I. paraguariensis* could modify the activity of this enzyme. Therefore, other behavioral and oxidative stress parameters were also evaluated considering the possible effects of* I. paraguariensis*.

## 2. Material and Methods

### 2.1. Chemical, Apparatus, and General Procedures

All chemical were of analytical grade. Acetonitrile, formic acid, gallic acid, chlorogenic acid, and caffeic acid were purchased from Merck (Darmstadt, Germany). Quercetin, theobromine, caffeine, rutin, catechin, and kaempferol were acquired from Sigma Chemical Co. (St. Louis, MO, USA). High performance liquid chromatography (HPLC-DAD) was performed with a Shimadzu Prominence Autosampler (SIL-20A) HPLC system (Shimadzu, Kyoto, Japan), equipped with Shimadzu LC-20AT reciprocating pumps connected to a DGU 20A5 degasser with a CBM 20A integrator, SPD-M20A diode array detector, and LC solution 1.22 SP1 software.

### 2.2. Quantification of Phenolics and Flavonoids Compounds by HPLC-DAD

Reverse phase chromatographic analyses were carried out under gradient conditions using C_18_ column (4.6 mm × 250 mm) packed with 5 *μ*m diameter particles. The mobile phase was water containing 1% formic acid (A) and acetonitrile (B), and the composition gradient was 13% of B until 10 min and changed to obtain 20%, 30%, 50%, 60%, 70%, 20%, and 10% B at 20, 30, 40, 50, 60, 70, and 80 min, respectively [16], with slight modifications.* I. paraguariensis* infusion was analyzed by dissolving in ethanol at a concentration of 20 mg/mL. The presence of nine antioxidants compounds was investigated, namely, gallic acid, chlorogenic acid, caffeic acid, catechin, quercetin, rutin, kaempferol, caffeine, and theobromine. Identification of these compounds was performed by comparing their retention time and UV absorption spectrum with those of the commercial standards. The flow rate was 0.7 mL/min, injection volume 50 *μ*L and the wavelengths were 254 nm for gallic acid, 270 nm for theobromine, 280 nm for catechin and caffeine, 327 nm for caffeic and chlorogenic acids, and 366 nm for quercetin, rutin, and kaempferol. All the samples and mobile phase were filtered through 0.45 *μ*m membrane filter (Millipore) and then degassed by ultrasonic bath prior to use. Stock solutions of standards references were prepared in the HPLC mobile phase at a concentration range of 0.045–0.300 mg/mL for kaempferol, quercetin, catechin, rutin, caffeine, and theobromine and 0.030–0.250 mg/mL for gallic, caffeic, and chlorogenic acids. The chromatography peaks were confirmed by comparing their retention time with those of reference standards and by DAD spectra (200 to 400 nm). Calibration curve for gallic acid was *Y* = 12539*x* + 1305.3 (*r* = 0.9997); catechin: *Y* = 12851*x* + 1289.5 (*r* = 0.9998); chlorogenic acid: *Y* = 13079*x* + 1195.8 (*r* = 0.9992); caffeic acid: *Y* = 11978*x* + 1326.2 (*r* = 0.9994); caffeine: *Y* = 13276*x* + 1293.6 (*r* = 0.9995); theobromine: *Y* = 12473*x* + 1275.8 (*r* = 0.9996); rutin: *Y* = 12763 + 1265.7 (*r* = 0.9999); quercetin: *Y* = 11780*x* + 1362.6 (*r* = 0.9995); and kaempferol: *Y* = 12583*x* + 1238.9 (*r* = 0.9997). All chromatography operations were carried out at ambient temperature and in triplicate. The limit of detection (LOD) and limit of quantification (LOQ) were calculated based on the standard deviation of the responses and the slope using three independent analytical curves. LOD and LOQ were calculated as 3.3 and 10 *σ*/S, respectively, where *σ* is the standard deviation of the response and S is the slope of the calibration curve [17].

### 2.3. Animals

Male Wistar rats (with 2 months of age) weighing 200 to 250 g from our breeding colony were kept in cages with five animals each with continuous access to food and water or infusion of* I. paraguariensis*. The room housing the cages was temperature-controlled (22 ± 2°C) and on a 12 h light/dark cycle with the lights going on at 7:00 a.m. Animals were maintained and used in accordance to the guidelines of the Brazilian Association for Laboratory Animal Science (Ethics Committee Approval number A011-09).

### 2.4. Experimental Design

*I. paraguariensis* was obtained from local supermarkets in a form of herb consumed by population. The extract was prepared as infusion like “chimarrão” or “mate.” Herbal commercial samples (25 g) were weighed and put into 500 mL of hot water (70°C) [12]. The infusion was filtered using filter paper and then cooled to room temperature. The yield of mate infusion was 30.45%. An aliquot of the infusion was subjected to qualitative analyses by HPLC. The extracts of mate were daily prepared and offered to the animals in place of drinking water during four weeks [12] ad libitum. Any difference of liquid ingestion was observed between the groups (data not shown). The dose of dry extract per rat was calculated with the values obtained in gravimetric assay and estimated at 2.31 g/kg/day. After four weeks, behavioral analysis of locomotor activity and anxiety was evaluated in animals receiving water (*n* = 11) or* I. paraguariensis* (*n* = 9). Our intent with elevated plus-maze and open field tests was to avoid any possible false positive result in the FST, since the alterations in locomotion or anxiety could modify the response of the animals in FST. After this evaluation, the group that received water was subdivided into two groups. One group of animals received two administrations of selegiline (10 mg/kg, i.p., dissolved in 0.9% NaCl), 24 hours and 30 minutes, before FST [18]. This group was the positive control to antidepressant activity and MAO inhibition. Thus, the experimental groups were named as: control (*n* = 6),* I. paraguariensis* (*n* = 9), and selegiline (*n* = 5). After the behavioral analysis, the animals were killed by decapitation and the brains were immediately excised and used in biochemical assays.

### 2.5. Behavioral Analysis

#### 2.5.1. Elevated Plus-Maze

To evaluate possible alterations in anxiety-like state caused by treatment with* I. paraguariensis*, animals were exposed to an elevated plus-maze apparatus [19, 20]. The percentage of time spent on open arm and the percentage of the entries into the open arms were calculated as follows: time spent or number of entries into the open arm/total time or total number of the entries into closed and open arms × 100, respectively.

#### 2.5.2. Open Field Test

To analyze possible changes in spontaneous locomotor and exploratory activity caused by treatment with* I. paraguariensis*, the animals were placed individually in the center of a circular open field arena divided into nine parts [21]. The effect of drugs on behavior was examined after* I. paraguariensis* treatment (on day 30). The number of rearing and the number of line crossings were measured over 5 min. Sections of open field test were evaluated 1 hour after elevated plus maze test.

#### 2.5.3. Forced Swimming Test

This experiment was performed using the FST according to the method previously published by Porsolt et al. [22, 23]. The effects of* I. paraguariensis* on FST were investigated after 24 hours and 4 weeks of treatment. Male rats were placed into a cylinder with a diameter of 40 cm containing a column of 17 cm of water at 27°C. The animals were trained 24 hours before the test for 5 min. Twenty-four hours later, the animals were exposed to the same experimental conditions for 5 min. A rat was judged to be immobile whenever it remained floating in the water, in an upright position, making only small movements to keep its head above the water. The immobility time was taken.

### 2.6. Tissue Preparations

Rats were killed about 1 hour after the last session of behavioral. The brains were immediately excised and put on ice. Then, they were homogenized in 10 volumes (w/v) of 10 mM Tris-HCl, pH 7.4. The homogenates were centrifuged at 4000 ×g for 10 min to yield a low-speed supernatant fraction (S1) that was used for the biochemical assays.

### 2.7. Biochemical Assays

#### 2.7.1. Lipid Peroxidation Assay

To evaluate the participation of lipid peroxidation in the action of* I. paraguariensis* or selegiline, thiobarbituric acid reactive species (TBARS) were determined as described by [24]. In brief, samples were incubated at 100°C for 1 hour in a medium containing 8.1% sodium dodecyl sulfate, 1.4 M acetic acid, pH 3.4, and 0.6% thiobarbituric acid. The pink chromogen produced in the reaction was measured spectrophotometrically at 532 nm. Results were expressed as nmol of TBARS/g of tissue.

#### 2.7.2. SH Levels

The total SH (TSH) and non-protein-SH (NPSH) content from samples were determined as described by [25]. For the nonprotein thiol groups (NP) determination, the samples of S1 were precipitated with 200 *μ*L of 10% trichloroacetic acid followed by centrifugation. The colorimetric assay was carried out in phosphate buffer 1 M, pH 7.4. The reaction was measured spectrophotometrically at 412 nm. Results were expressed as *μ*g/g of tissue.

#### 2.7.3. Vitamin C Levels

Cerebral vitamin C (ascorbic acid (AA)) levels were determined as described by Jacques-Silva et al. [26]. Brain homogenates were precipitated with 1 volume of 10% trichloroacetic acid followed by centrifugation. An aliquot of 300 *μ*L of the supernatants was mixed with 2,4-dinitrophenylhydrazine (4.5 mg/mL), CuSO_4_ (0.075 mg/mL), and trichloroacetic acid 13.3% (final volume 1 mL) and incubated for 3 h at 37°C. Then 1 mL of H_2_SO_4_ 65% (v/v) was added to the medium. The ascorbic acid levels were measured spectrophotometrically at 520 nm and calculated using a standard curve (1.5–4.5 *μ*M ascorbic acid freshly prepared in sulfuric acid).

#### 2.7.4. MAO Activity Assay

MAO activity was determined by measuring the kynuramine oxidation to 4-hydroxyquinoline [27–29]. The samples were preincubated at 37°C for 10 min with the irreversible and selective inhibitor clorgyline (250 nM) or pargyline (250 nM) to assay MAO-A or MAO-B activity, respectively. After 10 min, kynuramine was added as a nonselective substrate at concentrations equal to the corresponding Km value (45 *μ*M for MAO-A and 30 *μ*M for MAO-B). The reaction was incubated during 30 min at 37°C. After this time, the reaction was stopped with trichloroacetic acid (TCA) 10%. The samples were centrifuged at 5.000 ×g for 5 min. It was added to supernatant 1 M NaOH. The reaction was measured by fluorimetric method, using 315 nm (excitation) and 380 nm (emission). The results are represented as fluorescence intensity/mg protein.

#### 2.7.5. Protein Quantification

The total protein content in homogenates (S1) was determined by the method of Lowry and Rosebrough [30], using bovine serum albumin as standard.

### 2.8. Statistical Analysis

Data were analyzed by unpaired *t*-test or one-way ANOVA, followed by Tukey's post hoc test when appropriate. Significance was considered when *P* < 0.05.

## 3. Results

### 3.1. HPLC Analysis

**Figure 1:**
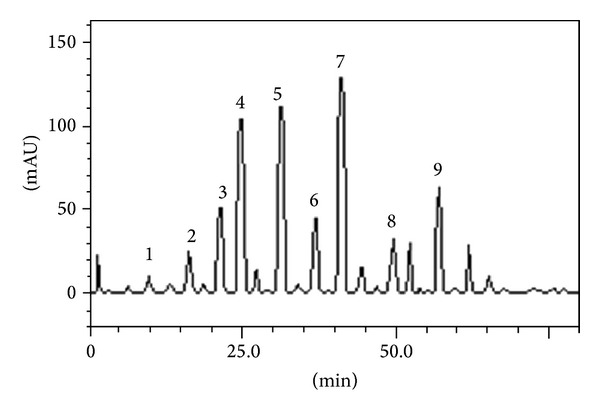
Representative high performance liquid chromatography profile of* Ilex paraguariensis* infusion,detection of UV was at 327 nm. Gallic acid (peak 1), catechin (peak 2), chlorogenic acid (peak 3), caffeic acid (peak 4), caffeine (peak 5), theobromine (peak 6), rutin (peak 7), quercetin (peak 8), and kaempferol (peak 9).

HPLC fingerprinting of* Ilex paraguariensis* infusion revealed the presence of the gallic acid (*t*_*R*_ = 10.17 min; peak 1), catechin (*t*_*R*_ = 16.23 min; peak 2), chlorogenic acid (*t*_*R*_ = 22.56 min; peak 3), caffeic acid (*t*_*R*_ = 24.97 min; peak 4), caffeine (*t*_*R*_ = 31.48 min; peak 5), theobromine (*t*_*R*_ = 37.12 min; peak 6), rutin (*t*_*R*_ = 41.73 min; peak 7), quercetin (*t*_*R*_ = 50.14 min; peak 8), and kaempferol (*t*_*R*_ = 57.39 min; peak 9) (Figure 1 and Table 1).

### 3.2. Effects of Treatment with* I. paraguariensis* on Elevated Plus-Maze Test in Rats

*I. paraguariensis* did not cause any significant effect on the number of head dipping, percentage of the time spent, and number of entries on the open arms of elevated plus-maze apparatus (Table 2).

### 3.3. Effects of Treatment with* I. paraguariensis* on Locomotor and Exploratory Activity in Rats

**Figure 2:**
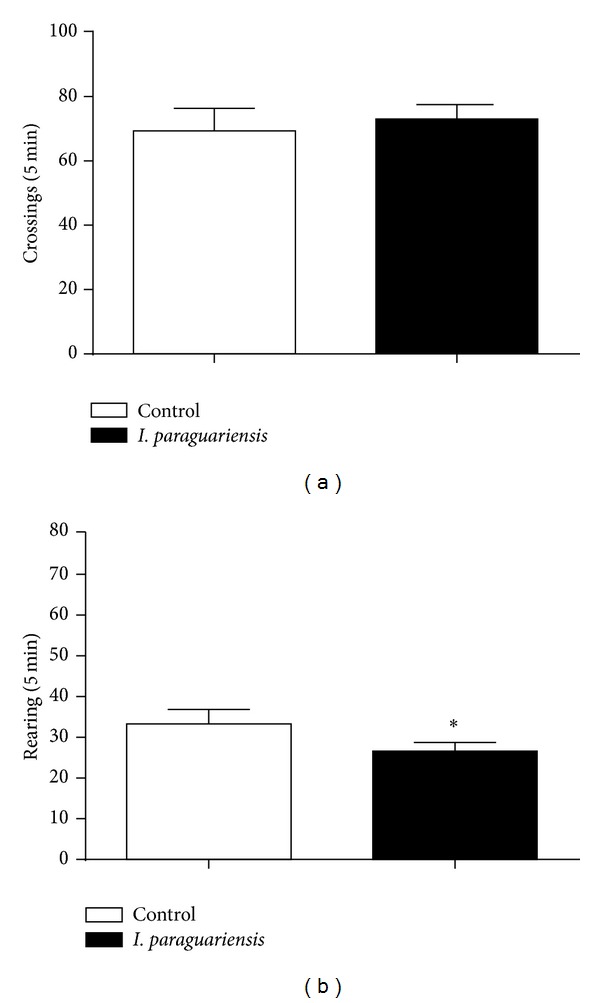
Effects of* I. paraguariensis* in the open field test in rats after 4 weeks of treatment. (a) Number of crossings and (b) rearing in 5 min. Values of number of crossings and rearing are represented by means ± SEM; control, *n* = 11;* I. paraguariensis*, *n* = 9. *Significant differences from control group.

*I. paraguariensis* treatment did not cause any effect on locomotor activity represented by number of crossings (Figure 2(a)). However, the number of rearing on the open field test was observed,* I. paraguariensis* caused a decrease in the number of rearing (*P* < 0.05; Figure 2(b)) in the open field test after 4 weeks of treatment.

### 3.4. Effects of Treatment with* I. paraguariensis* on Forced Swimming Test in Rats

**Figure 3:**
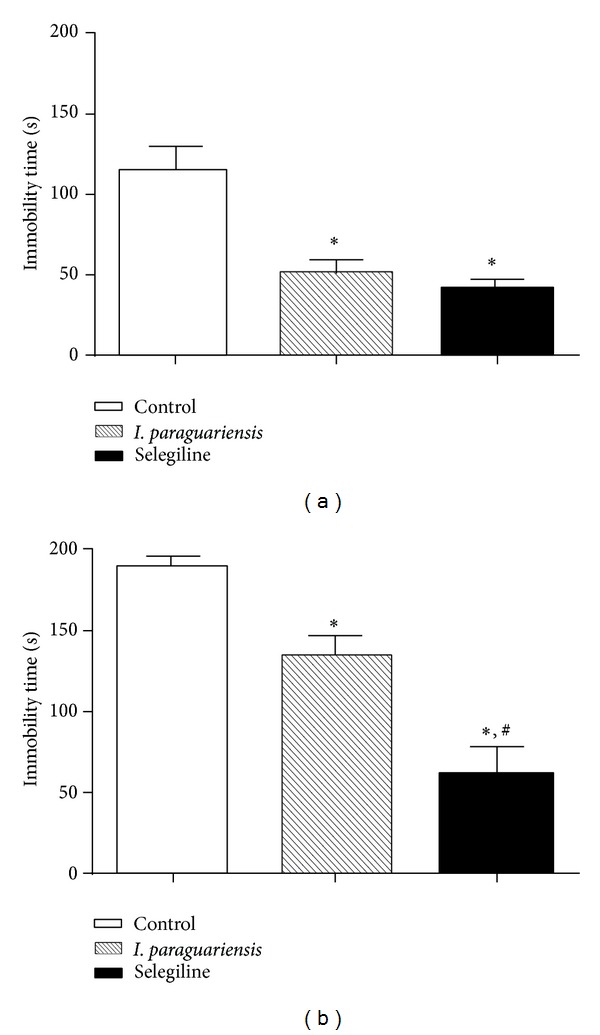
Effects of* I. paraguariensis* and selegiline on forced swimming test after (a) 24 hours and (b) 4 weeks of treatment with* I. paraguariensis* or two administrations (24 hours and 30 min before the test) of selegiline (10 mg/kg; i.p.). Values of immobility time are represented by means ± SEM (a); control, *n* = 4;* I. paraguariensis*, *n* = 5; and selegiline, *n* = 5; (b) control, *n* = 6;* I. paraguariensis*, *n* = 9; and selegiline, *n* = 5 (one-way ANOVA followed by Tukey's multiple comparison test). * represents significant differences from control group and ^#^ represents significant differences from* I. paraguariensis* group.

**Figure 4:**
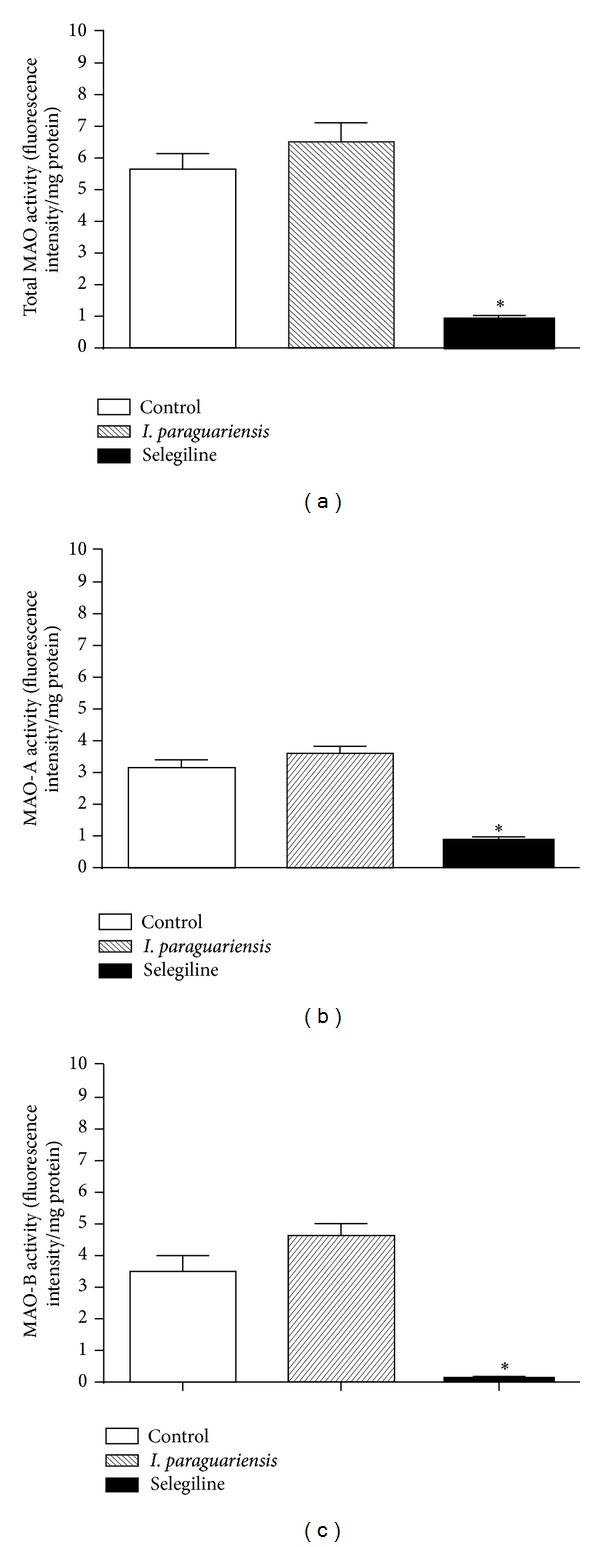
Effects of* I. paraguariensis* or selegiline on monoamine oxidase activity after 4 weeks of treatment with* I. paraguariensis* or two administrations (24 hours and 30 min before the test) of selegiline (10 mg/kg; i.p.). Values represent means ± SEM; control, *n* = 6;* I. paraguariensis*, *n* = 9; and selegiline, *n* = 5. * represents significant differences from control group and* I. paraguariensis* group.

To verify the possible antidepressant-like effects of* I. paraguariensis*, the FST was used. Statistical analyses revealed that aqueous extract of* I. paraguariensis* caused a significant reduction of immobility time in FST in relation to control group either when administered during 24 hours (*F*(2,11) = 18.73, *P* < 0.05; Figure 3(a)) or 4 weeks (*F*(2,19) = 25.45, *P* < 0.05; Figure 3(b)). Furthermore, selegiline, a MAO inhibitor, caused a significant reduction in immobility time in relation to control and* I. paraguariensis* group in this test (*P* < 0.05; Figure 3).

### 3.5. Effects of* I. paraguariensis* on MAO-A and MAO-B Activity

Since we detected a reduction in immobility in forced swimming test in rats treated with* I. paraguariensis*, we verified if this effect was caused by a possible inhibitory effect of* I. paraguariensis* on MAO-A or MAO-B activity (Figure 4). Selegiline administration was a positive control and caused a significant reduction in MAO-B activity (*F*(2,11) = 78.80, *P* < 0.05; Figure 4(c)). However, the activities of either MAO-A or MAO-B were not modified after treatment with* I. paraguariensis* (Figure 4).

### 3.6. Effects of* I. paraguariensis* on Oxidative Stress Parameters

As* I. paraguariensis* and selegiline are known by their antioxidant activity, we carried out assays for lipid peroxidation, levels of nonprotein SH, and ascorbic acid to verify possible changes in oxidative stress parameters that could modulate the MAO activity or could be a signal of protection/toxicity of both tested drugs. However, there was no significant difference among the groups in TBARS, nonprotein SH, and ascorbic acid levels in rats under treatment with* I. paraguariensis* (Table 3).

## 4. Discussion

The present study aimed to evaluate the possible antidepressant-like effect of* I. paraguariensis* in rats. We demonstrated that the treatment with* I. paraguariensis* decreased the immobility time on FST at 24 hours and this effect was the same after four weeks; a behavioral parameter was used to investigate the antidepressant potential of the drugs. Furthermore, the reduction in time of immobility by* I. paraguariensis* on FST was not associated with effects neither on locomotor nor on anxiolytic/anxiogenic activity. The antidepressant-like effect of* I. paraguariensis* was not associated with inhibitory effects on monoamine oxidase activity.

The Literature showed that* I. paraguariensis* has stimulant effects on central nervous system. Thus, the purpose of this study was to evaluate the antidepressant-like effect of* I. paraguariensis*. Despite previous some studies show the antidepressant-like activity of flavonoids [31, 32] which are present in the extract of* I. paraguariensis*, any study has evaluated the possible antidepressant-like activity of it. The present study showed that* I. paraguariensis* significantly reduced the immobility time on FST either after 24 hours or four weeks of treatment. HPLC analysis revealed the presence of caffeine in our extract. The percentage of caffeine was a little lesser than other ones [33, 34]. These differences can be attributed to the method of extraction and the different trademarks used in these studies. Previous studies demonstrated that caffeine exerts stimulant effects on motor activity of mice and rats [35]. Thus, in order to exclude a false positive, we use the open field test to verify possible alterations in locomotor activity in rats caused by* I. paraguariensis*. The treatment did not cause any effect on locomotor activity represented by the number of crossings, but when the number of rearing on the open field test was observed,* Ilex paraguariensis* caused a decrease in this number. These data can suggest that other compounds present in extract are able to decrease the number of rearing since caffeine has no effect on the central control of vertical activity [36]. In the same way, we evaluated if the presence of stimulants compounds like caffeine and theobromine in the extract of* I. paraguariensis* could cause anxiety. Furthermore, some studies have demonstrated that antidepressant agents can also possess anxiolytic properties in different anxiety animal models [36–38]. Thus, we investigated if* I. paraguariensis* treatment exerts anxiety or anxiolytic activity by using the plus-maze test. However, any significant effect was observed on elevated plus-maze test.

Monoamine oxidase is an enzyme responsible for degradation of monoamines, such as serotonin, dopamine, and norepinephrine. Moreover, the abnormal activity of the enzyme has been implicated in pathophysiology of depression [39–41]. The inhibition of MAO activity promotes an increase in monoamines and is one important target for the treatment of depression. Monoamine oxidase inhibitors have been used for decades in the treatment of depression and their antidepressant properties result from selective MAO-A inhibition in the central nervous system, which could lead to increased brain levels of 5-HT, NE, and DA [42, 43]. Recently, selegiline, an MAO-B inhibitor, has been used with success in the treatment of patients that are refractory to other antidepressant in a pharmaceutical transdermal preparation to avoid food interactions [44, 45]. We also investigated the involvement of the MAO in the possible antidepressant-like effect of* I. paraguariensis.* In the present study, MAO activity was not modified after treatment with* I. paraguariensis*. Thus, the antidepressant-like effect of* Ilex paraguariensis* represented by decrease in immobility time on FST seems not to be associated with MAO inhibition. Natural products have demonstrated numerous benefits in several animal models suggesting that antioxidant compound could exert protective effects [46–50]. However, there are studies showing that depending on the dose of the antioxidant compounds used, they could be prooxidants, causing toxicity and modulation in MAO activity by redox alteration [51, 52]. Selegiline was used as a positive control in our study because it possesses both MAO inhibitory activity and antioxidant properties [53]. We did not observe significant difference in oxidative stress parameters in this model. However, as we used a total brain homogenate in our study, some effects could better appear if they were analyzed in brain specific region.

## 5. Conclusion

In conclusion, the present study showed that* Ilex paraguariensis* presents an important effect on reducing immobility time on forced swimming test which could suggest an antidepressant-like effect of this extract. This effect is not associated with MAO inhibition or alterations in the parameters of oxidative stress. Furthermore, the extract did not show anxiolytic or anxiogenic activity. However, additional studies are needed to better understand the action mechanism of* I. paraguariensis*.

## Figures and Tables

**Table 1:** Composition of  *Ilex paraguariensis *infusion.

Compounds	*Ilex paraguariensis *		LOD *μ*g/mL	LOQ *μ*g/mL
	mg/g	%		
Gallic acid	0.65 ± 0.01^a^	0.06	0.016	0.052
Catechin	1.74 ± 0.03^b^	0.17	0.029	0.095
Chlorogenic acid	5.31 ± 0.01^c^	0.53	0.008	0.027
Caffeic acid	12.26 ± 0.03^d^	1.22	0.035	0.115
Caffeine	13.47 ± 0.01^d^	1.34	0.015	0.049
Theobromine	5.03 ± 0.01^c^	0.50	0.007	0.023
Rutin	17.82 ± 0.03^e^	1.78	0.026	0.086
Quercetin	3.16 ± 0.01^b^	0.31	0.032	0.104
Kaempferol	7.58 ± 0.02^f^	0.75	0.019	0.063

Results are expressed as mean ± standard deviations (SD) of three determinations.

Averages followed by different letters differ by Tukey's test at *P* < 0.05.

**Table 2:** Effects of  *I. paraguariensis* on elevated plus-maze test in rats during 5 minutes.

	Head dipping (5 min)	Entry into open arms (%)	Time spent into open arms (%)
Control	6.07 ± 1.60	17.93 ± 4.32	9.67 ± 3.40
*I. paraguariensis *	6.13 ± 0.96	18.10 ± 3.19	14.91 ± 5.31

Values are represented as means ± SEM (control, *n* = 11; *I. paraguariensis*, *n* = 9).

**Table 3:** Effects of  *I. paraguariensis* or selegiline treatments on oxidative stress parameters.

	TBARS (nmol of MDA/g tissue)	Nonprotein—SH (*μ*mol/g tissue)	Vitamin C (*μ*g of ascorbic acid/g tissue)
Control	148.30 ± 26.78	6.33 ± 1.22	608.70 ± 73.30
*I. paraguariensis *	157.60 ± 14.83	5.99 ± 0.61	643.10 ± 27.09
Selegiline	210.20 ± 44.01	3.69 ± 0.23	739.20 ± 47.80

Mean ± SEM; with control, *n* = 6; *I. paraguariensis*, *n* = 9; selegiline, *n* = 5.
